# Cancer Incidence and Epidemiological Trends in Punjab: A Population-Based Registry Analysis for State-Level Health Policy

**DOI:** 10.7759/cureus.87339

**Published:** 2025-07-05

**Authors:** Gurvinder S Grewal, Sumit Gupta, Manjinder S Sidhu, Gurpreet Brar, Sumeet Jain, Kunal Dhall, Kunal Jain, Shuchita Pathak, Akanksha Chhabra

**Affiliations:** 1 Medical Oncology, Dayanand Medical College and Hospital, Ludhiana, IND; 2 Surgical Oncology, Dayanand Medical College and Hospital, Ludhiana, IND; 3 Radiation Oncology, Dayanand Medical College and Hospital, Ludhiana, IND; 4 Medical Oncology, Dayanand Medical College and Hospital (DMCH) Cancer Care Center, Ludhiana, IND

**Keywords:** cancer epidemiology, incidence, mortality, population-based cancer registry, punjab

## Abstract

Objective: This study aimed to analyze cancer incidence and mortality patterns based on population-based cancer registry data for a defined region in Punjab, India, covering the period from January 2012 to December 2016.

Materials and methods: Active hospital and laboratory surveillance and community health worker monitoring identified cases, which were validated using CanReg5 for accuracy. We determined age-adjusted incidence rates (AARs) together with crude rates (CRs) and mortality-to-incidence ratios (M/I %).

Results: A total of 11,471 new cancer cases were recorded, comprising 5,394 (47.0%) men and 6,077 (53.0%) women. The AARs were 108.2 per 100,000 in men and 124.6 per 100,000 in women. The overall CR was 92.7 per 100,000, and the M/I % was 26.9%. The most common cancer sites were the esophagus (987; 18.3%) in men and breast (1,489; 24.5%) in women. Rural residents had higher M/I % (28.5%) than urban residents (24.3%; p = 0.003), indicating later-stage diagnosis and care barriers. The Indian registries recorded the highest incidence of childhood lymphoma among girls.

Conclusion: The findings demonstrate the immediate requirement for specific awareness programs and early detection initiatives that should focus on rural regions to decrease Punjab's cancer statistics.

## Introduction

Cancer poses an escalating public health challenge in India, with an estimated 1.4 million new cases and 880,000 deaths recorded in 2020, figures that have risen by over 14% compared to 2010 [1]. To monitor these trends, the Indian Council of Medical Research's National Cancer Registry Programme (NCRP) operates hospital-based and population-based registries, yet substantial regional heterogeneity remains under-explored [2].

Punjab accounts for just 2.3% of the national population but has only recently begun to generate systematic registry data through the Patiala Population-Based Cancer Registry (PBCR), established in 2012 to capture incident cancers via active surveillance of hospitals, diagnostic laboratories, and community health workers [3]. Although early PBCR bulletins have hinted at rising age-adjusted incidence rates (AARs) for breast and esophageal cancers, comprehensive analyses of mortality-to-incidence ratios (M/I %) and site-specific trends are lacking.

Despite growing concern over Punjab’s cancer burden, no study has yet leveraged PBCR data to quantify incidence rates, M/I %, and cancer site distribution in a manner that can directly inform state-level health policy. Therefore, we aimed to analyze all incident cancers registered in the Patiala PBCR from 2012 to 2016 to: (i) calculate age-adjusted and crude incidence rates stratified by sex and age; (ii) determine overall and rural-urban M/I %; and (iii) describe the most common cancer sites and benchmark these against national data. We hypothesized that Punjab’s overall cancer incidence exceeds national averages and exhibits distinct site-specific patterns, underscoring the need for tailored early-detection and prevention initiatives.

## Materials and methods

Study design and ethics approval

This retrospective, descriptive observational study included all incident cancer cases registered in the Patiala Population-Based Cancer Registry (PBCR) from January 1, 2012, to December 31, 2016. The protocol was approved by the Institutional Ethics Committee at Dayanand Medical College & Hospital (Approval No. IEC/DMCH/2025/045), and a waiver of informed consent was granted for the use of de-identified registry data.

Registry data and case selection

Incident cases were identified through active surveillance of all government and private hospitals (including AYUSH centers), diagnostic laboratories, and community health workers within the Patiala district [4]. We included all microscopically confirmed malignant neoplasms in residents diagnosed during the study period. Benign tumors, non-resident cases, and duplicate records were excluded. Data underwent validation and deduplication using CanReg5 software [5].

**Figure 1:**
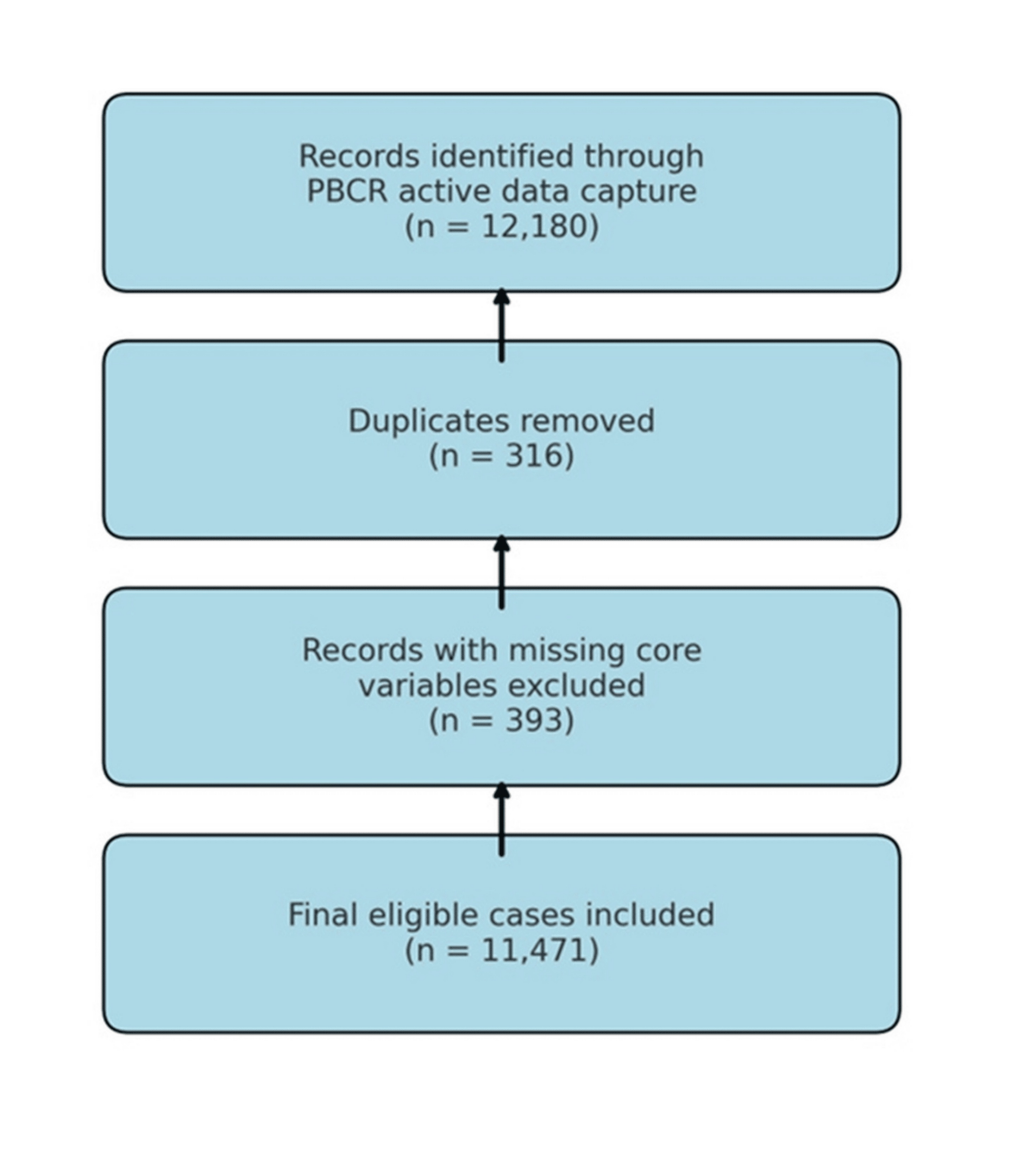
PRISMA-Style Case Selection Flow for Patiala PBCR (2012–2016) The figure outlines the case inclusion process from the Patiala Population-Based Cancer Registry (PBCR). Out of 12,180 records initially identified, 316 duplicates and 393 incomplete entries were excluded, resulting in 11,471 final eligible cases for analysis PRISMA: Preferred Reporting Items for Systematic Reviews and Meta-Analyses

Variables and endpoints

The primary outcome was the AAR, calculated by the direct method using the Segi world standard population [6]. Secondary outcomes included the crude incidence rate (CR), M/I %, and site-specific frequencies. The M/I % was calculated as the number of cancer deaths divided by the number of new cancer cases for the same time period, expressed as a percentage. This metric serves as a proxy for cancer survival in settings where individual-level survival data is not available. A higher M/I % indicates poorer outcomes, potentially due to late-stage presentation, aggressive tumor biology, or limited access to treatment. Rural versus urban residence was defined per the Census of India classification to compare M/I % across settings.

Data collection and quality assurance

Trained registry staff abstracted data following NCRP guidelines [4]. Quality assurance measures included periodic audits of completeness, validity, and comparability, with any discrepancies reconciled through hospital record review. Records missing key variables (age, sex, site, or vital status) were queried and updated when possible. The data abstraction process followed standardized procedures that met NCRP guidelines [4]. The case forms received two levels of review, starting with registry personnel abstraction, followed by supervisor verification. The CanReg5 software system detected inconsistent variables, which were resolved through source record re-checks. The core variables (age, sex, primary site, and vital status) achieved completeness rates above 99% while the post-audit reconciliation revealed less than 0.5% duplicate records. The site-specific case capture rates were validated through regular checks of hospital discharge records and pathology registry documents. Previous studies have emphasized the importance of robust regional cancer registries to track epidemiological trends and enable state-level planning [7,8].

Statistical analysis

All analyses were performed using IBM SPSS Statistics for Windows, Version 28 (Released 2021; IBM Corp., Armonk, New York, United States) [9]. Incidence rates are expressed per 100,000 population. Differences in M/I ratios between rural and urban areas were tested using chi-square tests, with a two-sided p < 0.05 indicating statistical significance. Missing data were handled via pairwise deletion.

## Results

Baseline characteristics

Between January 1, 2012 and December 31, 2016, the Patiala PBCR registered 11,471 new cancer cases: 5,394 (47.0%) in men and 6,077 (53.0%) in women, yielding a female-to-male ratio of 1.13:1. The registry covers an estimated population of 3.7 million, with a rural-to-urban population split of approximately 65:35 (Table 1).

**Table 1:** Demographic Profile of Registered Cancer Cases in Punjab (2012–2016) This table provides a summary of key demographic characteristics of cancer cases recorded in the Punjab Population-Based Cancer Registry from 2012 to 2016. It includes the total number of registered cases, sex distribution with percentages, female-to-male ratio, covered population size, and rural-to-urban population distribution.

Characteristic	Value
Total registered cases	11,471
Male, n (%)	5,394 (47.0%)
Female, n (%)	6,077 (53.0%)
Female-to-male ratio	1.13:1
Covered population	3.7 million
Rural-to-urban ratio	65:35

Incidence and mortality rate

The study population shows a significant difference between cancer occurrence rates and survival results between men and women according to Table 2 and Figure 1.

**Table 2:** Cancer Incidence Rates and Mortality-to-Incidence Ratios by Sex in Punjab (2012–2016) This table presents age-adjusted and crude cancer incidence rates per 100,000 population, along with mortality-to-incidence (M/I) ratios (%) for men, women, and overall populations, as reported by the Punjab Population-Based Cancer Registry (2012–2016). Age-adjusted rates are shown separately for men and women, while the crude incidence rate is provided for the overall population. All values are reported with their 95% confidence intervals (CI).

Metric	Male	Female	Overall
Age-adjusted incidence rate (per 100,000)	108.2 (95% CI: 104.7–111.7)	124.6 (95% CI: 121.1–128.1)	—
Crude incidence rate (per 100,000)	—	—	92.7 (95% CI: 91.0–94.4)
Mortality-to-incidence ratio (%)	30.3 (95% CI: 29.0–31.6)	23.9 (95% CI: 22.7–25.1)	26.9 (95% CI: 26.0–27.8)

**Figure 2:**
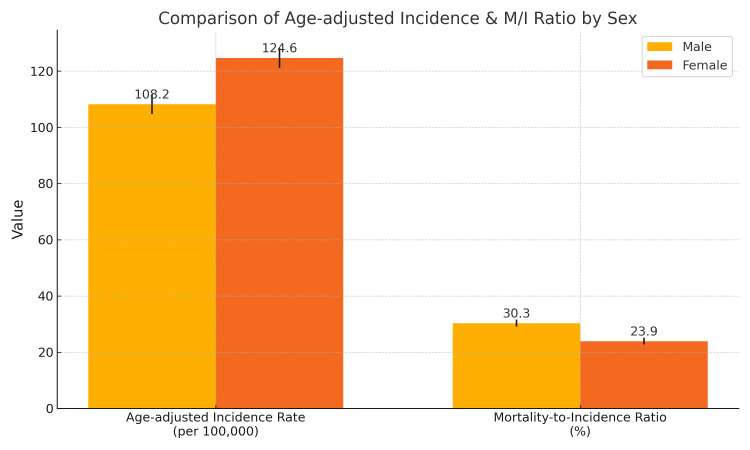
Comparison of Age-Adjusted Cancer Incidence Rates and Mortality-to-Incidence Ratios by Sex in Punjab (2012–2016). The figure displays age-adjusted incidence rates (per 100,000 population) and mortality-to-incidence (M/I) ratios (%) for male and female cancer patients, as recorded by the Punjab Population-Based Cancer Registry from 2012 to 2016. Error bars represent 95% confidence intervals. Female rates were higher for incidence (124.6 per 100,000) compared to male patients (108.2 per 100,000), whereas male patients exhibited a higher M/I ratio (30.3%) than female patients (23.9%).

The AAR showed higher numbers among women which indicates cancer affects them more during the study period. The M/I % was significantly lower among women than men even though women had higher cancer incidence rates which suggests earlier detection or different cancer aggressiveness, or better treatment access. The M/I % rate indicates a major potential for better cancer survival results in the region because male mortality rates exceed their incidence rates. The observed patterns demonstrate the necessity for specific early-detection and treatment approaches that should target male patients and cancer sites with high mortality rates. Compared with national GLOBOCAN 2022 estimates [1], Punjab exhibited significantly higher M/I ratios (p < 0.001).

Site distribution

In men, the leading cancer sites were the esophagus 987 (18.3%), lung 766 (14.2%), and oral cavity 685 (12.7%). In women, breast 1,489 (24.5%), cervix uteri 961 (15.8%), and ovary 541 (8.9%) predominated (Table 3). Among female patients, esophageal (213 cases, 3.5%), lung (195 cases, 3.2%), and oral cavity cancers (182 cases, 3.0%) were less frequent and did not rank among the top three sites. These patterns differ from national registry data [4], where lung and oral cavity cancers dominate in men and cervical cancer ranks second in women.

**Table 3:** Most Common Cancer Sites by Sex in Punjab (2012–2016) This table displays the leading cancer sites among men and women as reported by the Punjab Population-Based Cancer Registry during 2012–2016. The data are presented as the number of cases (n) with corresponding percentages (%) relative to the total cancer cases within each sex. The esophagus, lung, and oral cavity were the most common sites in men, while the breast, cervix uteri, and ovary were predominant in women.

Site	Male n (%)	Female n (%)
Esophagus	987 (18.3%)	—
Lung	766 (14.2%)	—
Oral cavity	685 (12.7%)	—
Breast	—	1,489 (24.5%)
Cervix uteri	—	961 (15.8%)
Ovary	—	541 (8.9%)

These patterns differ from national registry data [4], where lung and oral cavity cancers dominate in men and cervical cancer ranks second in women.

Subgroup analyses

Rural residents had a higher M/I % 3,279 deaths (28.5%, 95% CI: 27.0-30.0%) than urban residents 1,626 deaths (24.3%, 95% CI: 23.0-25.6%; χ² = 8.74, p = 0.003), indicating later stage at diagnosis and barriers to early care (Table 4).

**Table 4:** Mortality-to-Incidence Ratios by Residence in Punjab (2012–2016) This table presents the mortality-to-incidence (M/I) ratios (%) with 95% confidence intervals (CI) for cancer patients residing in rural and urban areas of Punjab, based on data from the Population-Based Cancer Registry (2012–2016). The p-value indicates a statistically significant difference in M/I ratios between rural and urban populations.

Residence	M/I % (95% CI)	p-value
Rural	28.5 (27.0–30.0)	0.003
Urban	24.3 (23.0–25.6)	0.003

Childhood cancer incidence

When stratified by age, the childhood cancer incidence increased from 3.5 per million in the 0-4-year group to 4.1 and 4.7 per million in the 5-9 and 10-14-year age groups, respectively. Lymphoma in girls was evenly distributed across age bands at approximately 1.2-1.3 per million and was the most frequent childhood cancer in girls with eight cases (3.7 per million), the highest rate reported across Indian PBCRs [4].

**Figure 3:**
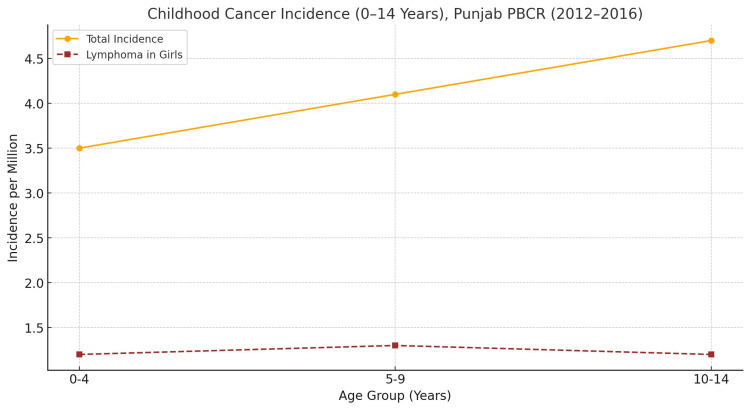
Childhood Cancer Incidence by Age Group (0–14 Years), Punjab PBCR (2012–2016) The line graph shows the total childhood cancer incidence (orange solid line) and lymphoma incidence in girls (red dashed line) across three age groups (0–4, 5–9, 10–14 years). The incidence is expressed per million population. A consistent but relatively high lymphoma rate among girls is observed compared to other PBCR reports from India.

**Figure 4:**
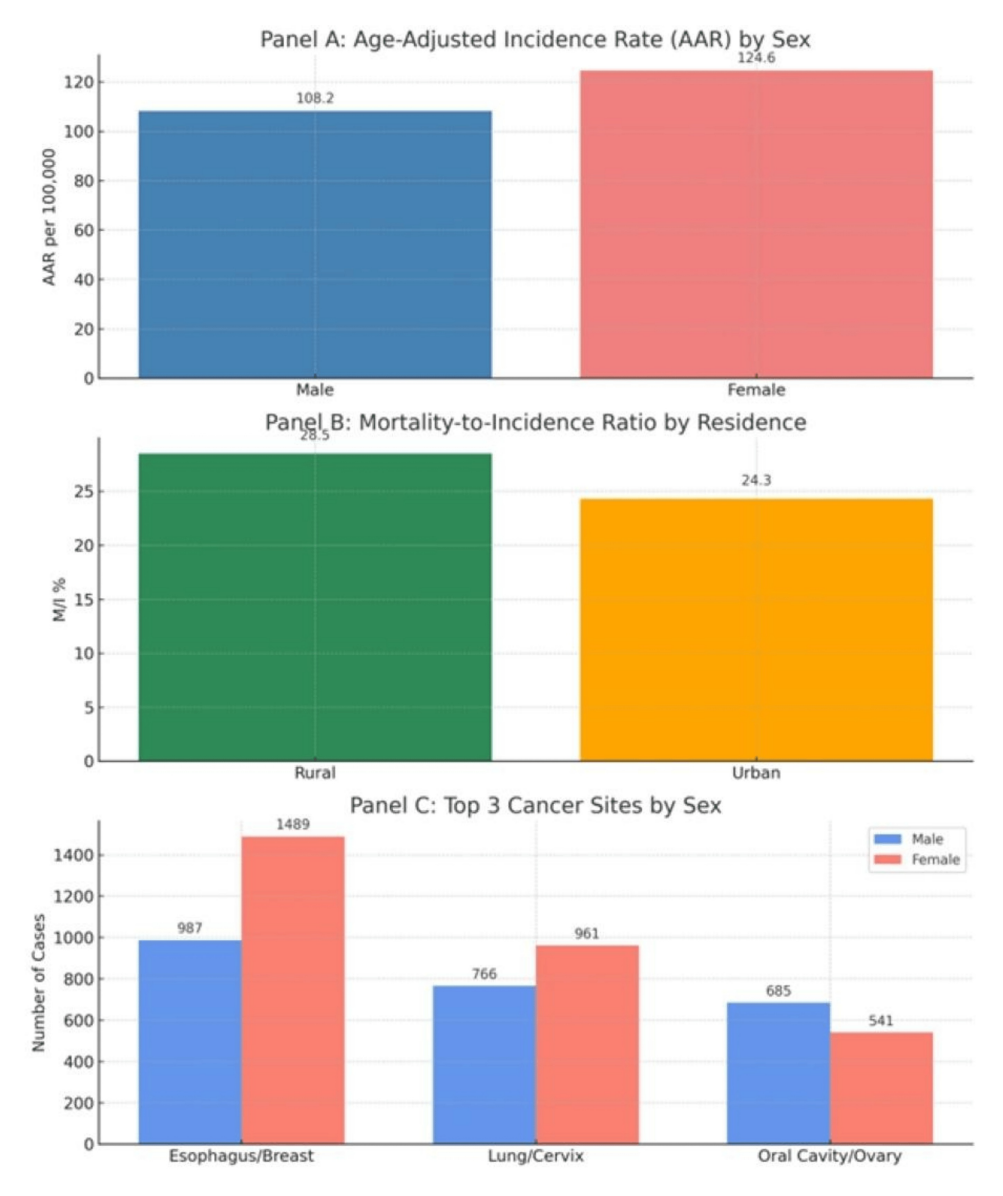
Combined Epidemiological Summary from Patiala PBCR (2012–2016) Panel A: Age-adjusted incidence rates (AAR per 100,000) are shown by sex. Women had a higher overall AAR (124.6) compared to men (108.2). Panel B: Mortality-to-incidence (M/I) ratios by residence, indicating a higher M/I% in rural areas (28.5%) than in urban areas (24.3%), suggesting disparities in early detection and treatment. Panel C: Distribution of the top three cancer sites by sex. Men predominantly presented with esophagus, lung, and oral cavity cancers, whereas women showed the highest numbers for breast, cervix, and ovary cancers. Bar values represent absolute case numbers or calculated rates as appropriate. Data derived from the Punjab PBCR registry (2012–2016)

A visual overview of the most relevant epidemiological findings is shown in Figure 4, including AARs by sex, M/I ratios by rural-urban residence, and top three cancer sites stratified by sex.

Data integrity

Data completeness for key variables (age, sex, primary site, vital status) exceeded 99%, and post-audit deduplication reduced duplicate records to under 0.5%.

## Discussion

The present study reveals that the AAR for women in Punjab is 124.6 per 100,000, approximately 21% higher than the national average of 102.9 per 100,000 reported by ICMR and GLOBOCAN data [1,2]. For men, the AAR was 108.2 per 100,000, exceeding the national male average by about 16%. The overall M/I % stands at 26.9%, with significantly higher mortality in the rural population (28.5%) compared to the urban population (24.3%; p = 0.003), indicating gaps in early detection and equitable care [6]. As illustrated in Figure 2, the higher AAR in women and disproportionate rural M/I ratios reinforce the urgent need for sex- and location-specific cancer control strategies. These findings are consistent with GLOBOCAN 2022 estimates, which report a national AAR of 102.9 per 100,000 [1]. Recent European registry data report rising incidence of breast and gastrointestinal cancers, underscoring common epidemiological patterns across regions [8]. Similar increasing trends for breast and esophageal cancers have also been observed in North India, particularly in Punjab and surrounding states [10]. The higher female AAR compared to most regional registries points to possible region-specific risk factors. While the dataset spans 2012-2016, it represents the most recent, validated, and comprehensive PBCR data available from Punjab as published by the National Centre for Disease Informatics and Research (NCDIR-ICMR) in 2020 [2]. Despite the data's age, comparison with national and international statistics underscores its continued relevance for informing cancer control initiatives. Earlier hospital-based reports from Punjab have documented a rising trend in breast and esophageal cancer since the early 2000s, consistent with our population-based data from 2012 to 2016. In comparison with other PBCRs across India, the female AAR of 124.6 per 100,000 in our study exceeds that of most northern and central registries, including Delhi (99.6), Bhopal (88.4), and Patna (91.2), as per NCRP 2020 [2]. Similarly, the M/I ratio in rural areas (28.5%) is notably higher than urban centers like Mumbai or Bengaluru, suggesting disparities in access and early detection. These inter-regional contrasts underscore the need for Punjab-specific cancer prevention and control strategies tailored to rural demographics. The pattern of cancer incidence may reflect a combination of lifestyle and environmental risk factors, including high tobacco use prevalence in rural areas, diets rich in nitrosamines, and exposure to pesticides and contaminated water [11]. These associations merit further exploration through analytical epidemiological studies to enable targeted prevention strategies [10]. The strength of this study lies in the use of high-quality registry data characterized by active case-finding, CanReg5-based deduplication, and rigorous audits, ensuring over 99% completeness of key variables. This methodological robustness enhances the reliability of our incidence and mortality estimates. However, limitations include the absence of staging and socioeconomic data, which constrain survival and disparity analyses. Future studies should aim to integrate clinical, environmental, and socioeconomic data to better elucidate causal factors and guide tailored public health measures. Our findings highlight the urgent need to strengthen early detection and community-based cancer screening, particularly in rural areas [12]. The WHO can achieve its goal of reducing premature cancer mortality by 2030 through expanded registry coverage and risk factor surveillance, which provides essential information for policy development and resource optimization [13]. The United States demonstrates through its data-driven national strategies that improved cancer outcomes can be achieved [14]. In addition, prioritizing rural health infrastructure and workforce development will be essential for closing equity gaps in cancer care delivery [15].

The PBCR dataset used (2012-2016) represents the most recently validated and publicly released data from Punjab by the NCDIR-ICMR as of 2020 [2]. The post-2016 data either have not been fully published or are still under validation and registry-level audits. The time lag reflects the quality assurance process-including verification, deduplication, and mortality linkage-essential for generating reliable cancer statistics in population-based registries. The M/I % is higher in rural areas (28.5% vs. 24.3%) because of delayed presentation, geographic inaccessibility of oncology services, and limited health-seeking behavior. These patterns underscore the critical need for rural-centric cancer control efforts encompassing community education, mobile screening units, and early referral pathways. The rural cancer registration process faces challenges because of weak infrastructure, which leads to under-reporting and misclassification of cases that stem from incomplete peripheral center data and limited access to histopathological confirmation. The reported mortality rates become higher than the actual incidence rates because of this issue. The PBCR datasets lacked staging and socioeconomic variables, which prevented us from conducting survival-adjusted or equity-focused analyses. The existing gaps in cancer registry data require immediate improvement, especially for low-resource areas.

## Conclusions

This study presents the first comprehensive PBCR-based analysis of cancer incidence in Punjab and highlights a significant burden among women and younger populations. These findings emphasize the urgent need for region-specific public health interventions to improve cancer screening, pediatric cancer care, and preventive measures.

The analysis benefits from high-quality registry data and active case validation, but the absence of staging and socioeconomic data limits the assessment of survival and disparities. Future research needs to integrate clinical and environmental data to identify causal factors and support precision prevention strategies. Enhancing early detection and improving equitable access to care, especially in underserved regions, are critical to mitigating the overall burden of cancer.
